# Down-Regulation of Nucleolar and Spindle-Associated Protein 1 (NUSAP1) Expression Suppresses Tumor and Cell Proliferation and Enhances Anti-Tumor Effect of Paclitaxel in Oral Squamous Cell Carcinoma

**DOI:** 10.1371/journal.pone.0142252

**Published:** 2015-11-10

**Authors:** Atsushi Okamoto, Morihiro Higo, Masashi Shiiba, Dai Nakashima, Tomoyoshi Koyama, Isao Miyamoto, Hiroki Kasama, Atsushi Kasamatsu, Katsunori Ogawara, Hidetaka Yokoe, Hideki Tanzawa, Katsuhiro Uzawa

**Affiliations:** 1 Department of Oral Science, Graduate School of Medicine, Chiba University, Chuo-ku, Chiba, Japan; 2 Department of Dentistry and Oral-Maxillofacial Surgery, Chiba University Hospital, Chuo-ku, Chiba, Japan; 3 Department of Medical Oncology, Graduate School of Medicine, Chiba University, Chuo-ku, Chiba, Japan; 4 Department of Oral and Maxillofacial Surgery Research Institute, National Defense Medical College Hospital, Tokorozawa-shi, Saitama, Japan; Istituto dei tumori Fondazione Pascale, ITALY

## Abstract

**Background:**

Nucleolar and spindle-associated protein 1 (NUSAP1) is an important mitotic regulator. In addition to its crucial function in mitosis, NUSAP1 has recently received attention due to the interesting roles in carcinogenesis. The aim of this study was to reveal functional mechanisms of NUSAP1 in oral squamous cell carcinoma (OSCC).

**Methods:**

mRNA and protein expression levels of NUSAP1 in 9 OSCC-derived cells were analyzed by quantitative reverse transcriptase-polymerase chain reaction (qRT-PCR) and immunoblotting analyses. The correlation between the NUSAP1 expression profile and the clinicopathological factors was evaluated by immunohistochemistry (IHC) in clinical OSCC samples (n = 70). The NUSAP1 knockdown cells were established with short hairpin RNA (shRNA) in OSCC cells, and functional assays were performed using these cells. In addition to the evaluation of cellular proliferation and cell cycle, we also investigated the potential role of NUSAP1 in paclitaxel (PTX)-induced cellular responses.

**Results:**

mRNA and protein expression of NUSAP1 were significantly up-regulated in OSCC-derived cells compared with human normal oral keratinocytes (*P* < 0.05). IHC revealed that NUSAP-1 expression is closely associated with primary advanced T stage (P<0.05). Suppression of NUSAP1 expression levels led to significant (*P* < 0.05) inhibition of cellular proliferation. Furthermore, apoptosis induced by PTX was enhanced in NUSAP1 knockdown OSCC cells.

**Conclusions:**

NUSAP1 may be a crucial biomarker for OSCC. Moreover, down-regulated NUSAP1 expression suppresses tumor proliferation and also enhances anti-tumor effect of PTX by activating apoptotic pathways. Thus, the present study strongly suggests that regulating NUSAP1 expression should contribute to the therapy for OSCC.

## Introduction

Oral squamous cell carcinoma (OSCC) is a frequently occurring neoplasm that is usually aggressive and has a poor prognosis, that accounts for 275,000 new cancer cases and more than 120,000 deaths annually [1]. Many risk factors have been identified, including human papillomavirus infection and tobacco and alcohol use [2–4]. However, some patients develop OSCC without risk factors, suggesting that host susceptibility plays an important role. Molecular changes in a number of oncogenes and tumor suppressor genes associated with the development of OSCC could be important clues for preventing this disease [2,5].

We previously reported gene expression profiling of OSCCs using microarray analysis to identify genes associated with oral carcinogenesis [6]. Of these, Nucleolar and spindle-associated protein 1 (NUSAP1) was detected as a significantly upregulated gene.

NUSAP1 is an important mitotic regulator and its activity is essential for a variety of cellular events that occur during mitosis ranging from spindle assembly to cytokinesis in addition to playing crucial roles during mitosis [6]. The process of cellular division must be carried out with high fidelity and minimal errors to ensure that at the end of every mitotic cycle, each daughter cell acquires an equal number of chromosomes. The deregulation of mitosis is a common feature of most cancers. NUSAP1 is a protein that is expressed selectively in proliferating cells. NUSAP1 depletion in cells causes G2/M arrest, abnormalities in interphase nuclei, abnormal chromosomal segregation, chromosomal misalignment, aberrant spindle assembly, defective cytokinesis, and decreased numbers of central spindle microtubules [6]. NUSAP1 is indispensable for mitosis.

The involvement of NUSAP1 in cancer has been reported in many recent studies. Overexpression of NUSAP1 has been observed in a variety of malignant tumors such as metastatic breast cancer [7], hepatocellular carcinomas [8], and pancreatic adenocarcinoma [9]. However, the expression and function of NUSAP1 in OSCCs have not been evaluated previously. According to our microarray data, NUSAP1 is considered to be associated with OSCC progression. In current study, we investigated the potential role of NUSAP1 in OSCC progression.

## Methods

### Ethics statement

The Ethics Committee of the Graduate School of Medicine, Chiba University (approval number, 236) approved the study protocol, which was performed according to the tenets of the Declaration of Helsinki. All patients provided written informed consent.

### OSCC-derived cell lines and tissue specimens

Immortalized human OSCC-derived cell lines (HSC-2, HSC-3, HSC-4, KOSC2, Ca9-22, Sa3, HO-1-N-1, HO-1-u-1, and KON) were obtained from the Human Science Research Resources Bank (Osaka, Japan) or the RIKEN BioResource Center (Ibaraki, Japan) through the National BioResource Project of the Ministry of Education, Culture, Sports, Science and Technology (Tokyo, Japan). Short tandem repeat profiles confirmed cellular identity. These cells were grown in Dulbecco’s modified Eagle medium/F-12 HAM (Sigma-Aldrich, St. Louis, MO, USA) supplemented with 10% fetal bovine serum (Sigma) and 50 units/ml penicillin and streptomycin (Sigma). Primary cultured human normal oral keratinocytes (HNOKs) were obtained from healthy oral mucosa epithelium specimens collected from young patients at Chiba University Hospital. Three independent HNOKs were primary cultured and maintained in oral keratinocyte medium (ScienCell Research Laboratories, Carlsbad, CA, USA) comprised of 5 ml of oral keratinocyte growth supplement (ScienCell Research Laboratories) and 5 ml of penicillin/streptomycin solution (ScienCell Research Laboratories) [10,11]. Three independent HNOKs were primary cultured and maintained in Oral Keratinocyte Medium (ScienCell Research Laboratories, Carlsbad, CA, USA).

Seventy pairs of primary OSCC samples and patient-matched normal epithelial specimens were obtained during surgeries performed at Chiba University Hospital. The resected tissues were fixed in 20% buffered formaldehyde solution for pathologic diagnosis and immunohistochemistry (IHC). Histopathologic diagnosis of each OSCC sample was performed according to the World Health Organization criteria by the Department of Pathology of Chiba University Hospital [12]. Clinicopathologic staging was determined by the tumor-node-metastasis classification of the International Union against Cancer [13]. All patients had histologically confirmed OSCC; tumoral samples were checked to ensure that tumoral tissue was present in more than 80% of specimens.

### Preparation of cDNA

Total RNA was isolated from OSCC-derived cell lines using Trizol Reagent (Invitrogen, Carlsbad, CA, USA) according to the manufacturer’s instructions. cDNA was generated from 5 μg of total RNA using Ready-To-Go You-Prime First-Strand Beads (GE Healthcare, Buckinghamshire, UK) and oligo (dT) primer (Sigma Genosys, Ishikari, Japan) according to the manufacturer’s instructions.

### mRNA expression analysis

Real-time quantitative reverse transcriptase-polymerase chain reaction (qRT-PCR) was performed using LightCycler 480 apparatus (Roche Diagnostics, Mannheim, Germany). Primers and universal probes were designed using the Universal Probe Library (Roche Diagnostics), which specifies the most suitable set. The primer sequences used for qRT-PCR were: NUSAP1, forward, 5’- CTGTGCTTGGGACACAAA-3’; reverse, 5’-TTGTCAACTTGAATGGGGTAATAA-3’; and universal probe #84., and the glyceraldehyde-3-phosphate dehydrogenase (GAPDH), forward, 5’-CATCTCTGCCCCCTCTGCTGA-3’; reverse, 5’-GGATGACCTTGCCCACAGCCT-3’; and universal probe #60. The transcript amount for NUSAP1 was estimated from the respective standard curves and normalized to the GAPDH transcript amount determined in corresponding samples. All samples were analyzed in triplicate, and three independent preparations of RNA were analyzed from each cell line.

### Immunoblotting analysis

The cells were washed twice with cold phosphate-buffered saline (PBS) and centrifuged briefly. The cellular pellets were incubated at 4°C for 30 minutes in a lysis buffer (7 M urea, 2 M thiourea, 4% w/v CHAPS, and 10 mM Tris [pH, 7.4]) with the proteinase inhibitor cocktail (Roche Diagnostics). The protein concentration was measured with a Bio-Rad Protein Assay (Bio-Rad Laboratories, Hercules, CA, USA). Protein extracts were separated by sodium dodecyl sulfate polyacrylamide gel electrophoresis (SDS-PAGE) in 4–12% gel, transferred to nitrocellulose membranes, and blocked for 1 hour at room temperature in Blocking One (Nacalai Tesque, Tokyo, Japan). The membranes were incubated with rabbit anti-NUSAP1 antibody (ProteinTech Group, Chicago, IL, USA) for 4 hours at room temperature. The membrane was washed with 0.1% Tween-20 in Tris-buffered saline, incubated with secondary antibody and coupled to horseradish peroxidase-conjugated antimouse IgG (Promega, Madison, WI, USA) for 1 hour at room temperature. The proteins were detected by SuperSignal Chemiluminescent substrate (Thermo, Waltham, MA, USA). Finally, the signals were visualized by exposing the membrane to a cooled CCD camera system (ATTO, Tokyo, Japan). Signal intensities were quantitated using the CS Analyzer version 3.0 (ATTO).

### IHC

IHC was performed on 4-μm sections of paraffin-embedded specimens using rabbit anti-NUSAP1 polyclonal antibody (ProteinTech Group). Briefly, after deparaffinization and hydration, the endogeneous peroxidase activity was quenched by a 30-minute incubation in a mixture of 0.3% hydrogen peroxide solution in 100% methanol; the sections were blocked for 2 hours at room temperature with 1.5% blocking serum (Santa Cruz Biotechnology) in PBS before reacting with the anti-NUSAP1 antibody (1:1000 dilution) at 4°C in a moist chamber overnight. Upon incubation with the primary antibody, the specimens were washed three times with PBS and treated with Envision reagent (DAKO, Carpinteria, CA, USA) followed by color development in 3,3’-diaminobenzidine tetrahydrochloride (DAKO). The slides then were counterstained with hematoxylin, dehydrated with ethanol, cleaned with xylene, and mounted. As a negative control, triplicate sections were immunostained without exposure to primary antibodies, to confirm the staining specificity. To quantify the status of NUSAP1 protein expression in those components, we used the IHC score systems described previously [14]. The intensities of the NUSAP1 immunoreactions were scored as follows: 1+, weak; 2+, moderate; and 3+, intense. The cellular numbers and staining intensities then were multiplied to produce a NUSAP1 IHC score. Cases with a NUSAP1 IHC score exceeding 73.0 (± 3 standard deviation score for normal tissue) were defined as NUSAP1-positive. The ±3 standard deviation cutoff, which statistically is just 0.2% of the measurement and expected to fall outside this range, was used because it is not likely to be affected by a random experimental error produced by sample manipulation [15]. Two independent pathologists from Chiba University Hospital, neither of whom had knowledge of the patients’ clinical status, made these judgments. To calculate the overall survival rate, we surveyed each patient’s life and month of death.

### Transfection with short hairpin RNA (shRNA) plasmid

OSCC cell line (HSC-3) were transfected with the NUSAP1 shRNA (shNUSAP1) or the Mock shRNA (shMock) vectors (Santa Cruz Biotechnology, TX, USA) using by Lipofectamine LTX and Plus Reagents (Invitrogen). After transfection, the stable transfectants were isolated by the culture medium containing 2 ng/ml puromycin (Invitrogen). Two to three weeks after transfection, viable colonies were transferred to new dishes, and shNUSAP1-transfected cell (shNUSAP1-HSC-3) and shMock-transfected cell (shMock-HSC-3) were established. Similarly, Sa3 cell line was transfected with NUSAP1 shRNA (shNUSAP1) or the Mock shRNA (shMock), and transfectants, shNUSAP1-Sa3 and shMock-Sa3, were established.

### Cellular proliferation assay

The NUSAP1 shRNA-transfected cells and control cells were seeded in six-well plates at a density of 1 × 10^4^ viable cells/well. Cells were harvested and counted every 24 hours up to 7 days. At the indicated time points, the cells were trypsinized and viable cell number was counted using a hemocytometer in triplicate samples.

### Cell Cycle Analysis

In order to synchronize cells at the G0/G1 or G2/M transition, cells were treated with 200 ng/ml nocodazole (Sigma) for 20 hours according to the previous reports [16,17]. Cell-cycle analysis was performed as described previously [10,18–20].

### Apoptosis assay

To analyze the apoptotic effects induced by PTX, the shNUSAP1 and Mock cells were incubated with PTX (100nM). Cell death was quantified using the FITC Annexin V Apoptosis Detection kit I (BD Bioscience, CA, USA), following the manufacturer’s instructions.

### Statistical analysis

Statistical significance was determined using the Mann-Whitney’s *U* test. The overall survival rate was evaluated using the log-rank test. P<0.05 was considered statistically significant. The data are expressed as the mean ± the standard error of the mean (SEM).

## Results

### Evaluation of NUSAP1 mRNA and protein expression in OSCC-derived cell lines

**Fig 1:**
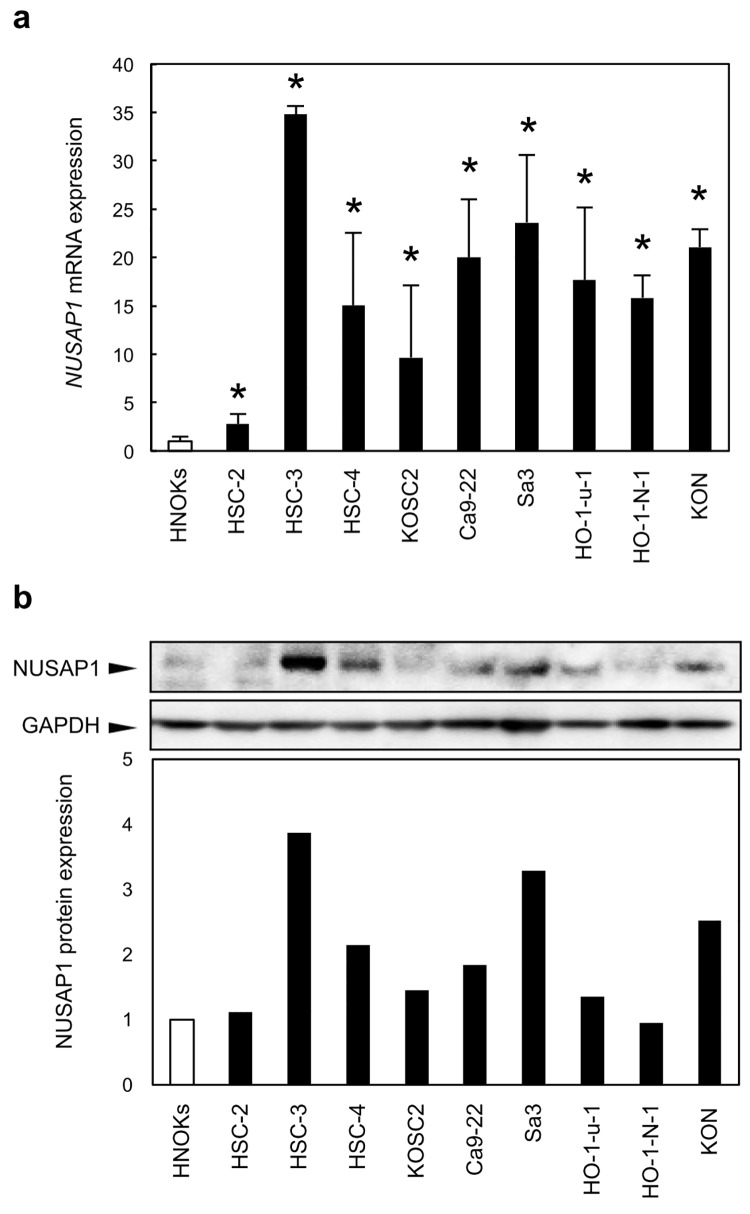
Evaluation of NUSAP1 expression in OSCC-derived cell lines. a. Quantification of *NUSAP1* mRNA expression levels in OSCC-derived cell lines by qRT-PCR analysis. mRNA expression levels are normalized to GAPDH. Significant (*p* < 0.05, Mann-Whitney’s *U* test*) up-regulation of *NUSAP1* mRNA is seen in nine OSCC-derived cell lines (HSC-2, HSC-3, HSC-4, KOSC2, Ca9-22, Sa3, HO-1-N-1, HO-1-u-1, and KON) compared with the HNOKs. Data are expressed as the means ± SEM of triplicate results. b. Representative immunoblotting data of NUSAP1 in OSCC-derived cell lines and HNOKs show that NUSAP1 protein expression is up-regulated in OSCC-derived cell lines compared with the HNOKs. The molecular weight of the NUSAP1 is 49 kDa. Densitometric NUSAP1 protein data are normalized to GAPDH protein levels. The values are expressed as a percentage of the HNOKs.

To investigate mRNA expression of *NUSAP1* identified as a cancer-related gene by our previous microarray data [14], we performed qRT-PCR analysis using 9 OSCC-derived cell lines and HNOKs. *NUSAP1* mRNA was up-regulated significantly (*p* < 0.05) in all OSCC-derived cell lines compared with the HNOKs (Fig 1A). We also performed immunoblotting analysis to investigate NUSAP1 protein expression status in the OSCC-derived cell lines and HNOKs (Fig 1B). A significant increase in NUSAP1 protein expression was observed in OSCC cell lines examined when compared with the HNOKs.

### Evaluation of NUSAP1 protein expression in primary OSCC

**Fig 2:**
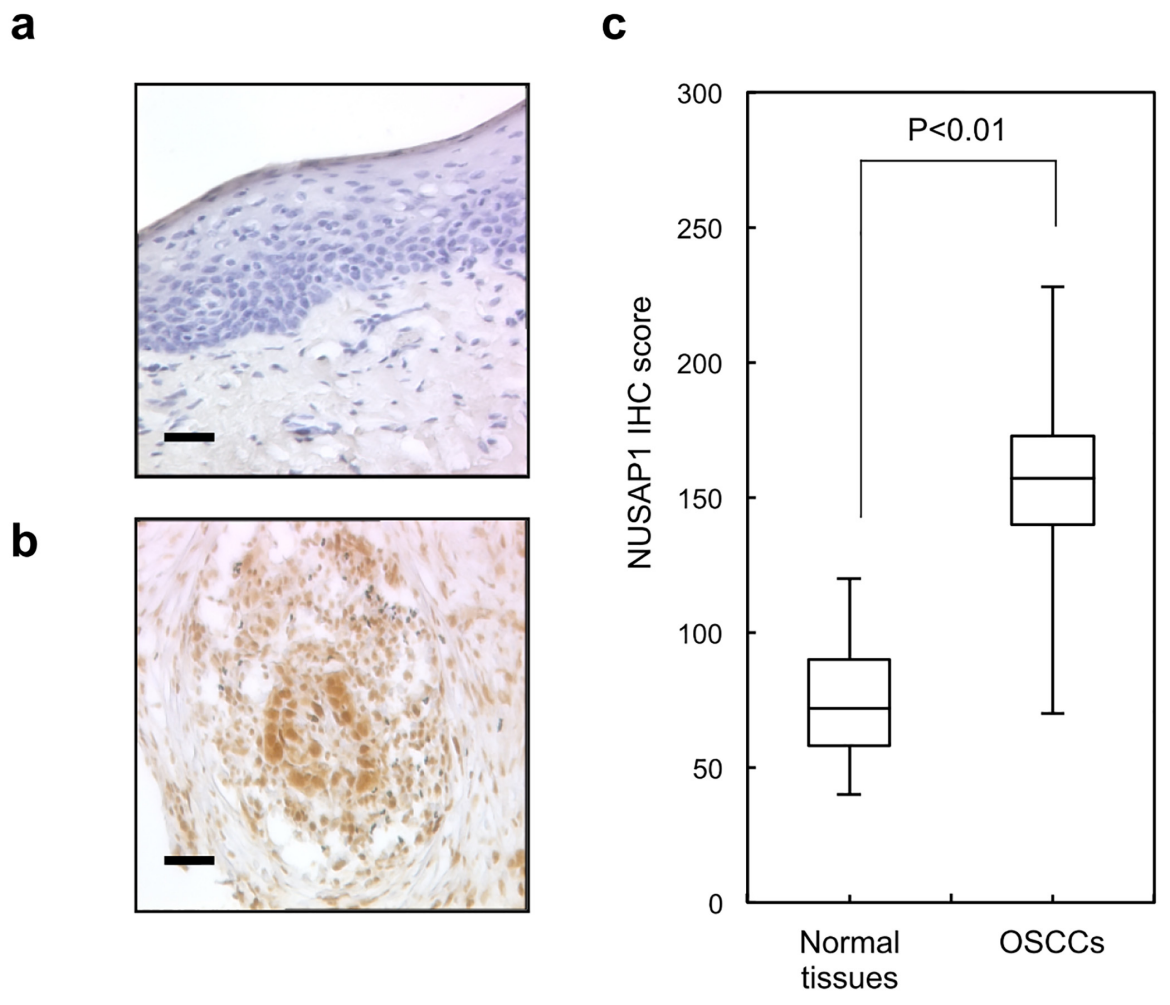
Evaluation of NUSAP1 protein expression in primary OSCCs. Representative IHC results for NUSAP1 in a normal oral tissue and b primary OSCC (original magnification, x400. Scale bars, 50 μm). Strong NUSAP1 immunoreactivity is detected in primary OSCCs, whereas normal oral tissues show almost negative immunostaining. c The state of NUSAP1 protein expression in the normal counterparts and primary OSCCs (n = 70). The NUSAP1 IHC scores for normal oral tissues and OSCCs range from 0 to 38.1 (median 12.8) and 40.2 to 170.3 (median 136.3), respectively. NUSAP1 protein expression levels in OSCCs are significantly (*p* < 0.001, Mann-Whitney’s *U* test) higher than that in normal oral tissues.

Representative IHC results for NUSAP1 expression in normal oral tissue and primary OSCC are shown in Fig 2A and 2B, respectively. Positive staining was seen predominantly in the nuclei of primary OSCC samples. The IHC scores of NUSAP1 for normal oral tissues and OSCCs ranged from 43.1 to 128.4 (median, 72.8) and 73.1 to 229.3 (median, 157.3), respectively (Fig 2C). NUSAP1 positive OSCCs were correlated significant (*p* < 0.05) difference was found in the NUSAP1 expression levels between the T1/T2 group and the T3/T4 group, indicating NUSAP1 expression levels are higher in advanced stages compared with early stages. The correlation between the clinicopathologic characteristics of the patients with OSCC and the status of NUSAP1 expression using the IHC scoring system is shown in Table 1. The overall survival rates in the NUSAP1-positive OSCCs (n = 54) and the NUSAP1-negative OSCCs (n = 16) were 87.3% and 85.2%, respectively. The survival rates in the NUSAP1-positive group were lower than in the NUSAP1-negative group, but the difference did not reach significance (P = 0.79) (S1 Fig).

**Table 1:** Correlation between NUSAP1 expression and parameters in OSCCs.

			Results of immunostaining				
			No. patients				
Clinical classification		Total	NUSAP1(-)		NUSAP1(+)		*p* value
Age at surgery (years)							
	<60	19	7	(35%)	12	(65%)	0.214
	60–70	27	5	(17%)	22	(83%)	
	70>	24	4	(20%)	20	(80%)	
Gender							
	Male	40	9	(25%)	31	(75%)	0.512
	Female	30	7	(20%)	23	(80%)	
T-primary tumor size							
	T1	5	1	(20%)	4	(80%)	0.204
	T2	37	12	(32%)	25	(68%)	
	T3	13	2	(10%)	11	(90%)	
	T4	15	1	(6%)	14	(94%)	
	T1+T2	42	13	(33%)	29	(67%)	0.048*
	T3+T4	28	3	(8%)	25	(92%)	
N-regional lymph node							
	Negative	39	11	(25%)	28	(75%)	0.533
	Positive	31	5	(21%)	26	(79%)	
TNM stage							
	I	5	1	(20%)	4	(80%)	0.691
	II	25	6	(24%)	19	(76%)	
	III	21	4	(25%)	17	(75%)	
	IV	19	5	(21%)	14	(79%)	
Histopathological type							
	Well-differentiated	43	9	(21%)	34	(79%)	0.456
	Moderately differentiated	16	5	(36%)	11	(64%)	
	Poorly differentiated	11	2	(22%)	9	(78%)	
Tumoral site							
	Gingiva	15	3	(20%)	12	(80%)	0.552
	Tongue	40	10	(25%)	30	(75%)	
	Buccal mucosa	6	1	(17%)	5	(83%)	
	Oral floor	9	2	(22%)	7	(78%)	
Total		70	16		54		

NUSAP1(-), down-regulated NUSAP1; NUSAP1(+), up-regulated NUSAP1.

*p < 0.05. (χ2 test).

### Establishment of NUSAP1 knockdown cells

**Fig 3:**
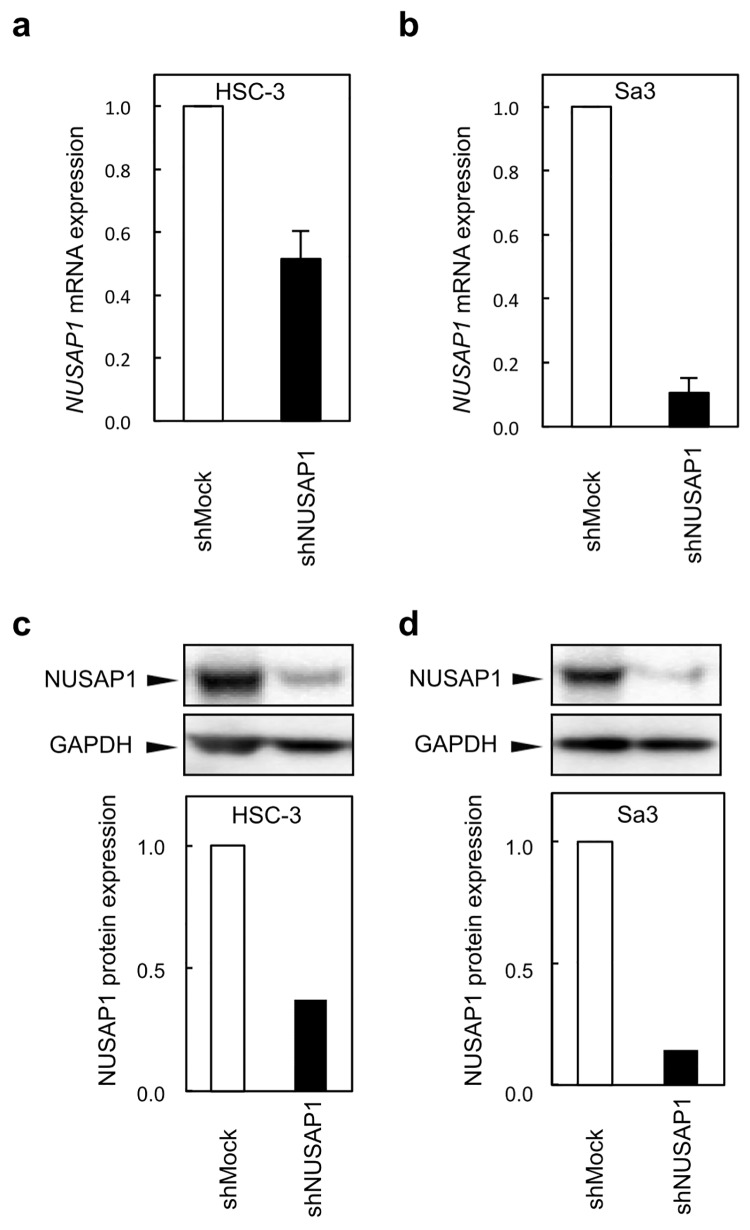
Establishment of NUSAP1 knockdown cells. To obtain stable NUSAP1 knockdown transfectants, we performed transfection of the shNUSAP1 and Mock vectors into OSCC cell lines (HSC-3 and Sa3). a,b Expression of *NUSAP1* mRNA in shNUSAP1 and Mock cells. c,d Immunoblotting analysis of NUSAP1 protein in shNUSAP1 and Mock cells. The NUSAP1 mRNA and protein are significantly down-regulated in shNUSAP1 cells (HSC-3 and Sa3).

qRT-PCR and immunoblotting analyses were performed to assess the efficiency of NUSAP1 knockdown in shRNA transfectants. *NUSAP1* mRNA expression in the shNUSAP1-HSC-3 and shNUSAP1-Sa3 cells was significantly (*p* < 0.05) lower than that in the shMock-HSC and shMock-Sa3 cells, respectively (Fig 3A). Consistent with the mRNA levels, NUSAP1 protein levels were markedly decreased in shNUSAP1-HSC-3 and shNUSAP1-Sa3 cells (Fig 3B).

### Reduced cellular proliferation in NUSAP1 knockdown cells

**Fig 4:**
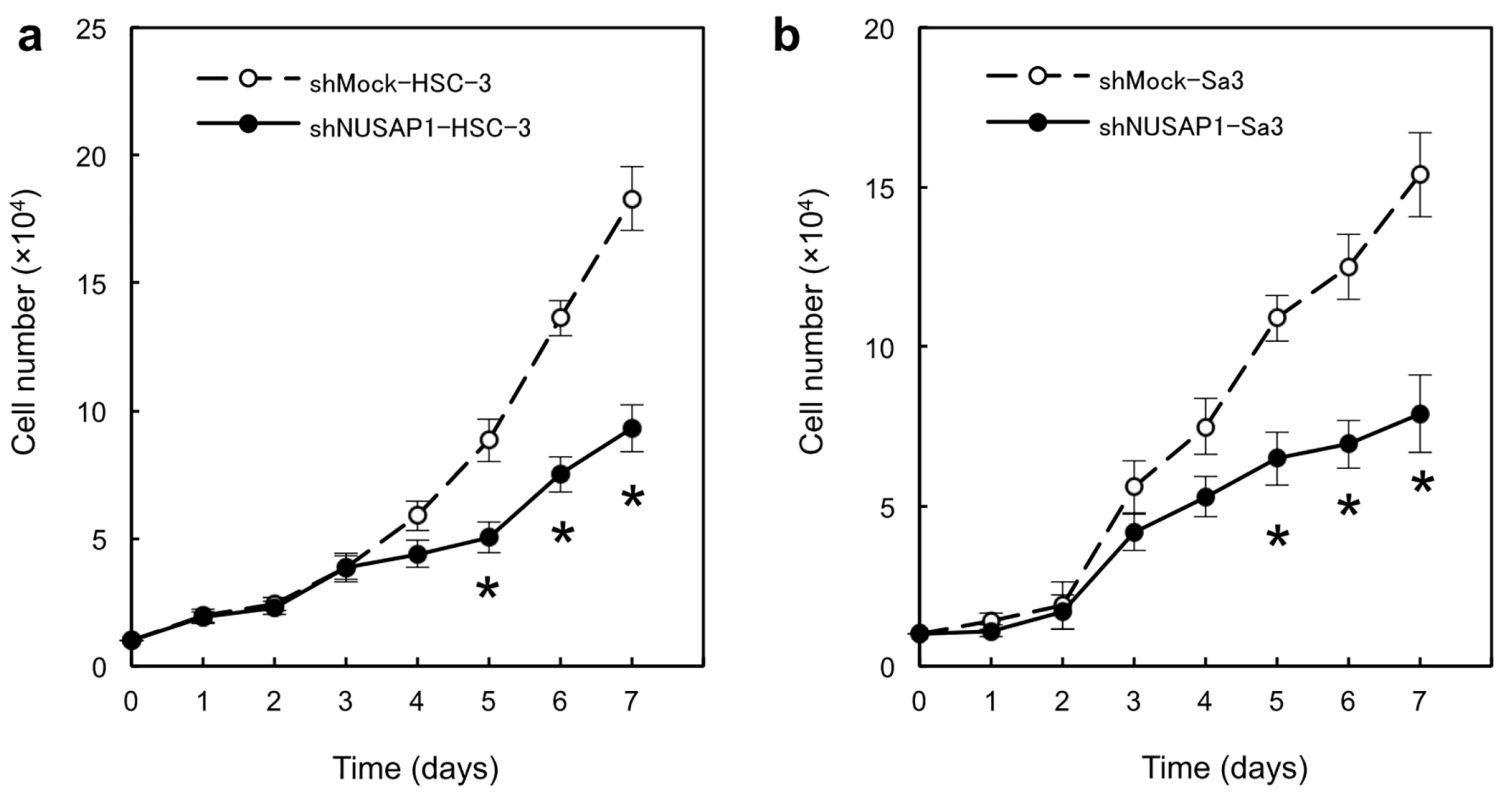
Functional analyses of NUSAP1 knockdown cells. Reduced cellular growth was observed in a shNUSAP1 HSC-3 and b shNUSAP1 Sa3 cells. To determine the effect of shNUSAP1 on cellular proliferation, shNUSAP1 and Mock cells are seeded in 6-well plates at a density of 1×10^4^ viable cells/well. The shNUSAP1 HSC-3 and shNUSAP1 Sa3 cells show a significant (*p* < 0.05, Mann-Whitney’s *U* test*) decrease in cellular growth compared with Mock cells. The results are expressed as the means ± SEM of values from three assays. The asterisks indicate significant (*p* < 0.05, Mann-Whitney *U* test*) differences between the shNUSAP1 and Mock cells. c shNUSAP1- and Mock-transfected cells were counted on 4 consecutive days by a hemocytometer.

Effect of downregulation of NUSAP1 on cellular proliferation was investigated. shNUSAP1-HSC-3 (Fig 4A) and shNUSAP1-Sa3 (Fig 4B) cells exhibited the significant decreased proliferation rates when compared with each control cells (*p* < 0.05).

### Cell-cycle analysis of shNUSAP1 cells

**Fig 5:**
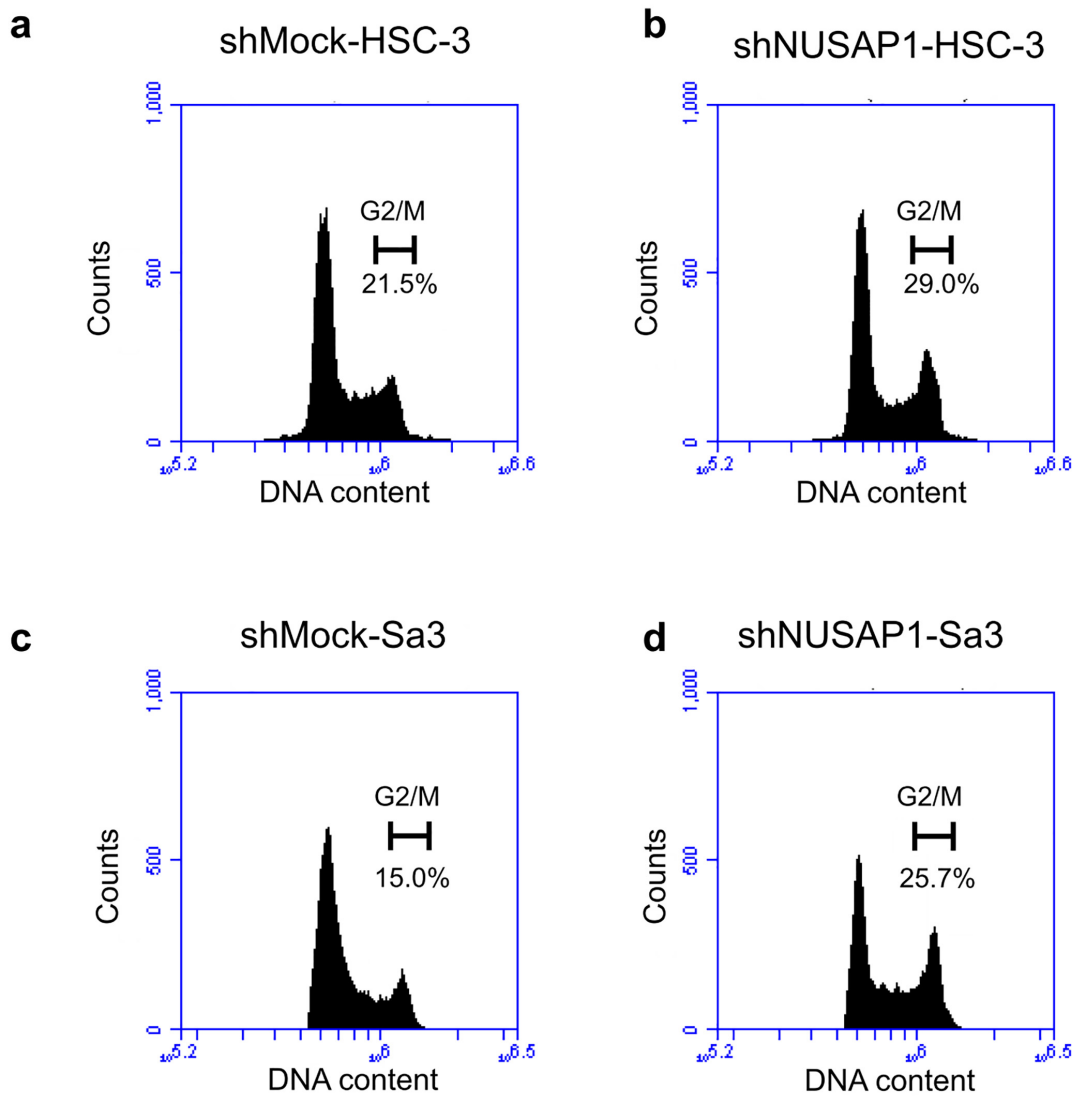
shNUSAP1 promotes G2 arrest. Flow cytometric analysis was performed to investigate cell-cycle progression in the shNUSAP1- and shMock-transfected cells after synchronization at the G2/M phase to treatment with nocodazole. a-d The percentage of cells at the G2 phase in the shNUSAP1-transfected cells (HSC-3- and Sa3-derived transfectants) is increased markedly compared with the shMock- transfected cells.

The percentage of the shNUSAP1 cells at the G2/M phase was higher than that of the shMock cells (Fig 5). The shNUSAP1-HSC-3 cells had 29% of G2/M phase cells, which was higher than the number of shMock-HSC-3 cells in the G2/M phase (21.5%). Similarly, shNUSAP1-Sa3 cells significantly induced G2/M phase arrest (25.7%). These results indicated that shNUSAP1 inhibited cellular proliferation by cell-cycle arrest at the G2/M phase.

### Combination of shNUSAP1 and paclitaxel (PTX) enhances apoptosis in OSCC cells

To examine whether NUSAP1 function affects paclitaxel-induced cell death of HSC-3 cells, we performed FACS analysis on HSC-3 cells that were transfected with Mock vector or shNUSAP1 and then treated with paclitaxel (100 nM, 48h).

**Fig 6:**
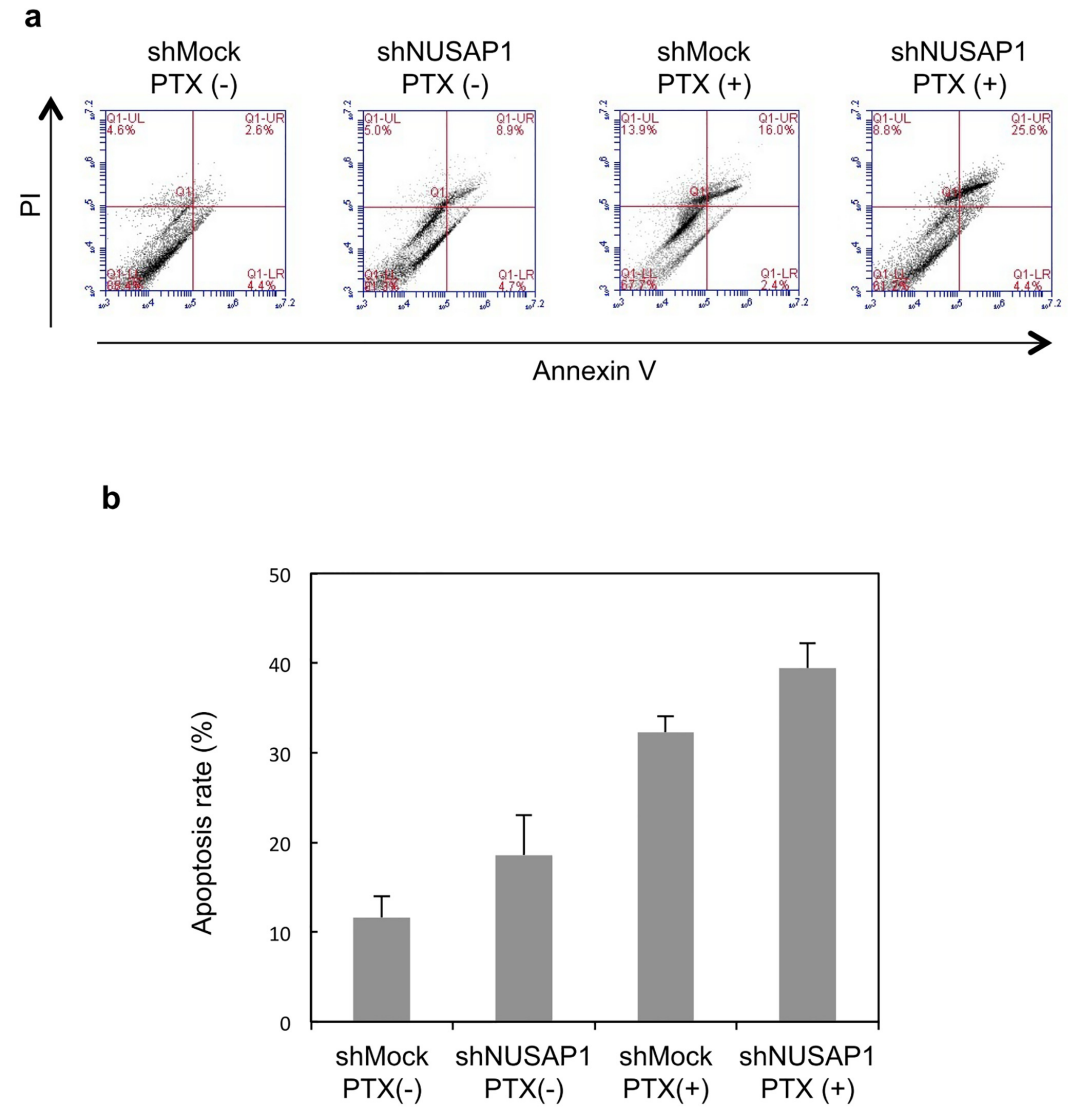
Apoptosis of shNUSAP1 determined by flow cytometry. a HSC-3 cells after dual staining with Annexin V-FITC and propidium iodide (PI). Annexin V-FITC/PI staining is monitored overtime, after 48 hours in HSC-3 and Sa3 cells exposed to PTX at concentrations corresponding to IC50 (100nM). Representative dot plots of three independent experiments are given, presenting intact cells in the lower-left quadrant, FITC(-)/PI(-); early apoptotic cells in the lower-right quadrant, FITC(+)/PI(-); late apoptotic or necrotic cells in the upper-right quadrant, FITC(+)/PI(+); and necrotic cells in the upper-left quadrant, FITC(-)/PI(+). b Total apoptosis rate was analyzed from 3 times experiment.

As shown in Fig 6, cells transfected with shNUSAP1 and treated with paclitaxel (shNUSAP1+paclitaxel) exhibited significantly higher rates of cell death, indicating increased sensitivity of HSC-3 cells to paclitaxel-induced apoptosis.

## Discussion

Although overexpression of NUSAP1 has been found in breast cancer [7], hepatocellular carcinoma [8] and pancreatic cancer [9], there is no study demonstrating the altered expression profile of NUSAP1 in oral cancer. Our previous DNA microarray analysis revealed the up-regulated expression of NUSAP1 in oral carcinoma [14], suggesting that NUSAP1 may be related to oral carcinoma. Thus, we investigated the expression profiles and biological functions of NUSAP1 to clarify the roles of NUSAP1 in oral carcinoma.

First, we checked the expression profiles of NUSAP1 in OSCC-derived cell lines and OSCC cases. The data showed that mRNA and protein expression of NUSAP1 were significantly up-regulated in the cell lines and the OSCC samples. Furthermore, overexpression of NUSAP1 was observed in the advanced tumor samples. These results indicate that the up-regulated expression of NUSAP1 should be closely associated with OSCC, especially in the accelerating cellular proliferation, and that NUSAP1 can be a clinically significant biomarker for OSCC.

Our data also revealed that cellular proliferation apparently decreased and the relative number of cells in G2/M phase was increased when the NUSAP1 expression was suppressed. The data suggest that overexpressed NUSAP1 induces dysregulated cell cycle leading to the active proliferation of tumor cells. So we assume that the accelerated tumor cell proliferation may be controlled by decreasing NUSAP1 expression.

PTX inhibits tumor cell division via microtubule stabilization induced by the microtubule depolymerizing inhibitory effect. In the meanwhile, NUSAP1 is also known as a microtubule binding and bundling protein [21,22]. Namely, PTX and NUSAP1 have the biological functions on the common target, microtubules. Raemaekers reported that the microtubules bundled with NUSAP1 are resistant to depolymerization by the microtubule-depolymerizing drug such as nocodazole [21]. Thus, it is reasonable to assume that down-regulated expression of NUSAP1 may enhance the PTX-induced cytotoxic effect such as apoptosis. The present study actually showed NUSAP1-knocked down cells exhibited higher sensitivity for PTX than parental cells. Moreover, the rate of G2/M cell cycle phase was relatively increased in the NUSAP1-knocked down cells. And PTX is known to induce cell cycle arrest in G2/M phase [23]. These facts strongly suggest that NUSAP1 has conflict functions on PTX-induced cytotoxicity. In other words, down-regulated NUSAP1 expression can enhance the anti-tumor activity of PTX with its synergistic effects, and might overcome chemoresistance to PTX in OSCCs. The present study firstly demonstrates the close relationship between NUSAP1 and PTX.

## Conclusions

The present study shows that NUSAP1 is overexpressed in OSCC and that the altered NUSAP1 expression profile is closely associated with tumor size in primary OSCC samples, suggesting that NUSAP1 may be a crucial biomarker for OSCC. Moreover, NUSAP1 knockdown led to suppressed cellular proliferation and enhanced PTX-induced apoptosis in OSCC cells. The present study demonstrates that regulating NUSAP1 expression should contribute to the therapy for OSCC.

## Supporting Information

S1 Fig Overall survival rate. The NUSAP1 expression level is not correlated significantly (P = 0.79, log-rank test) with overall survival. The overall survival rate in the NUSAP1-positive OSCCs (n = 54) and the NUSAP1-negative OSCCs (n = 16) are 87.3% and 85.2%, respectively.
(TIF)

S2 Fig Effect of NUSAP1 gene silencing on PTX sensitivity in human cancer cell lines as determined by MTS assay. NUSAP1 was knocked down in HSC-3, and sensitivity to PTX was determined by MTS assay. Cells treated with non-targeting gene were used as a control. IC50 values were determined by the MTS assay.
(TIF)

S1 Table Information on the cancer cell lines. (TIF)

## Abbreviations

OSCC: oral squamous cell carcinoma
NUSAP1: Nucleolar and spindle-associated protein 1
PTX: paclitaxel
